# Shape Stability of Metallic Nanoplates: A Molecular Dynamics Study

**DOI:** 10.1186/s11671-019-3192-7

**Published:** 2019-11-29

**Authors:** Xiwen Chen, Rao Huang, Tien-Mo Shih, Yu-Hua Wen

**Affiliations:** 1 Department of Physics, Xiamen University, Xiamen, 361005 China; 2 Department of Mechanical Engineering, University of California, Berkeley, CA 94720 USA

**Keywords:** Metal, Nanoplate, Stability, Molecular dynamics

## Abstract

Metallic nanoplates have attracted widespread interests owing to their functional versatility, which relies heavily on their morphologies. In this study, the shape stability of several metallic nanoplates with body-centered-cubic (bcc) lattices is investigated by employing molecular dynamics simulations. It is found that the nanoplate with (110) surface planes is the most stable compared to the ones with (111) and (001) surfaces, and their shapes evolve with different patterns as the temperature increases. The formation of differently orientated facets is observed in the (001) nanoplates, which leads to the accumulation of shear stress and thus results in the subsequent formation of saddle shape. The associated shape evolution is quantitatively characterized. Further simulations suggest that the shape stability could be tuned by facet orientations, nanoplate sizes (including diameter and thickness), and components.

## Introduction

As an important class of functional nanomaterials, metallic nanoplates have been extensively synthesized and examined owing to their excellent activities in catalysis [1–4], tunable optical properties [1, 5–7], potential utilization in microcircuits, among others [8, 9]. As is well known, the application performance of nanomaterials strongly depends on their structures and morphologies. Therefore, an in-depth understanding of the shape stability of these metallic nanoplates should be an issue of crucial importance to their synthesis and usage. From the thermodynamics point of view, the structure of a single nanoplate deviates from the energetic minimum and is supposed to develop into a sphere-like nanoparticle owing to the tendency of minimizing its surface energy. However, as metastable configurations, nanoplates are not rarely observed in experiments because of the participation of complex kinetic factors [10]. Relatively higher temperature increases the possibility for the system to escape the kinetic trapping and to realize the state with lower energy. Especially for those nanoplates with thicknesses as thin as several atomic layers, the significantly high surface-to-volume ratios imply a large proportion of atoms with weak bonding (i.e., high mobility), which are sensitive to the ambient environment and hence are presumably easy to induce the shape change. Induced by the adjusted external conditions, the transformations of shape and structure have been observed to occur in the solid regime [11–15]. Note that this kind of transformations is not uniquely determined by the thermodynamics to develop towards the more energetically favored spheres, while the reversed pathway from spherical particles to the anisotropic plates, similar to the Ostwald ripening, has also been discovered to be triggered by the thermal treatment [11].

Experimental investigations have been carried out to examine the thermal properties of metallic nanoplates. For examples, in situ transmission electron microscope (TEM) results combined with other structural analyses show that Au nanoplates present (110) facets around the edges consisting of the most unstable atoms, and fragmentation occurs upon heating [15]. Nevertheless, the microscopic experiment finds it extremely difficult to provide quantitative characterizations of the shape evolution mechanism. Alternatively, molecular dynamics (MD) simulation, as an ideal tool, can present direct images about the shape transformations at the resolution of atomic level. Accordingly, we performed the MD calculations on metallic nanoplates to disclose their morphology change mechanisms by describing the curving and buckling process involved. Metals with body-centered-cubic (bcc) lattices are addressed because relevant understanding on their shape stability is still lacking despite their common existence in experiments [16]. This study serves as a reference to both the synthesis and applications of these metallic nanoplates.

## Methods

Iron (Fe) nanoplates with bcc lattices (lattice constant *a* = 2.8665 Å), which are composed of three atomic layers (the diameter *d* = 32*a* in most cases), were first modeled. Initially, their surfaces are respectively set to be different low-index planes, including (111), (001), and (110). In addition, other bcc metallic nanoplates, such as W, Nb, Mo, and Cr, were also accordingly constructed. These models were created via commands in the MD package LAMMPS [17].

The interatomic interactions were described by the corresponding embedded atom method (EAM) potentials [18–22]. The initial models were first quasi-statically relaxed to a local minimum energy state through the conjugate gradient method (CGM). After full relaxation, continuous heating was simulated in a canonical (NVT) ensemble using LAMMPS, and the quantities of state (energy and stress tensor) are correspondingly exported. The temperature was set to increase from 1 to 300 K (or higher) with an increment of 1 K. Under a timestep of 2 fs, the relaxation time of 200 ps at each temperature is employed, and the statistically averaged quantities are taken from the last 8 ps. The uncertainty of the simulations mainly comes from two aspects: the accuracy of the potentials and the convergency of the energy at each temperature. Note that the potentials we used have been widely adopted in molecular simulations and repeatedly verified [23–27], mean while 200 ps is examined to be sufficient allowing the system to reach its thermal equilibrium, we hence believe our simulations are reliable.

In addition, the local stress tensor of the i_th_ atom was calculated by

$$ {\sigma}_{\alpha \beta}=\frac{1}{2{\Omega}_i}\sum \limits_{j\ne i}{F}_{ij}^{\alpha }{R}_{ij}^{\beta }, $$

in which *α* and *β* could be *x*, *y*, and *z*; *F*_*ij*_ and *R*_*ij*_ are the force and distance between atoms *i* and *j*, respectively [28]. Ω_*i*_ is the local volume that can be identified with the volume of the Voronoi polyhedra constructed by the perpendicular planes that bisect the lines between atom *i* and all its neighbor atoms, which has been calculated through the equal volume method [29].

## Results and Discussion

**Fig. 1:**
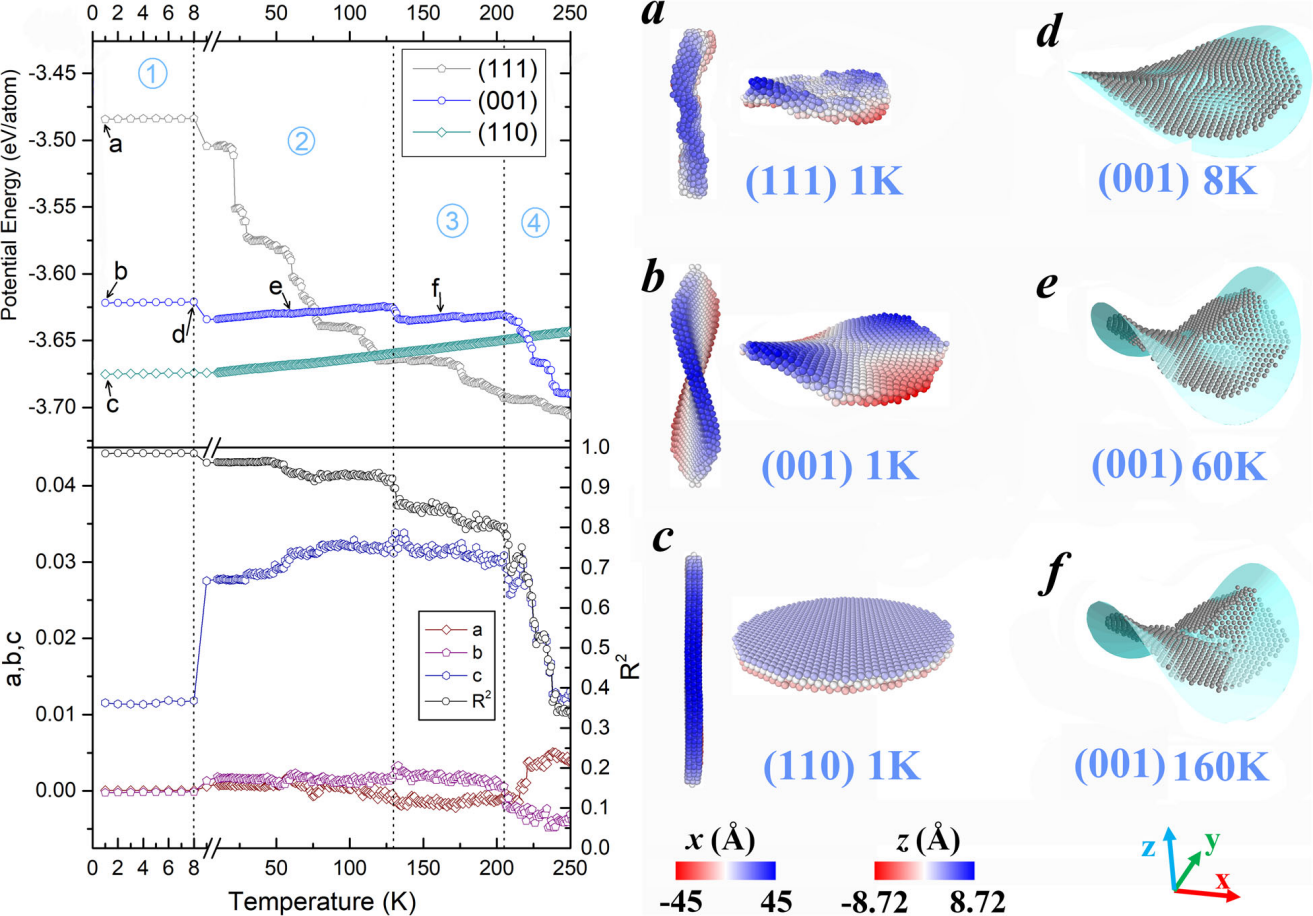
Left panel: Temperature evolution of the potential energy of three nanoplates (upper plots), and the geometric parameters obtained by fitting the middle layer of the (001) nanoplate (lower plots); Right panel: **a**, **b**, **c** Snapshots of three nanoplates after relaxation. **d**, **e**, **f** Snapshots of the middle layer in the (001) nanoplate taken at representative temperatures. Cyan surfaces denote the corresponding fitting results

As the temperature rises, the morphologies of the three Fe nanoplates evolve with different patterns. The upper plots in the left panel of Fig. 1 display their temperature dependent potential energies (*E*_p_). For the three nanoplates, crystal planes with different Miller indices lead to a clear hierarchy in structural stability. According to the calculations, the averaged potential energies per atom (not shown in Fig. 1) are − 2.833, − 3.457, and − 3.668 eV/atom respectively for the initial configurations with (111), (001), and (110) surfaces. Considering that the nanoplates are as thin as three atomic layers, it is natural to find that their energy values are in the same order of the surface energies of the three corresponding crystal planes (2.58, 2.47, and 2.37 J/m^2^ for (111), (001), and (110) surfaces, respectively [30]). With distinctively higher potential energies, the nanoplates with flat (111) and (001) crystal planes are not able to maintain their initial structures as constructed. They immediately transform into metastable states with curved surfaces (cf. snapshots (a) and (b) in the right panel of Fig. 1). In contrast, the (110) nanoplate presents the best structural stability. Its morphology (see Fig. 1c) keeps invariant throughout the entire temperature region examined, which can be corroborated by the energy steadily increasing with a linear trend. As for the other two nanoplates, their shape deformations exhibit different features. The least stable (111) nanoplate turns into an irregular form instantly after relaxation (see Fig. 1a), and this irregular geometry facilitates the progress of shrinking into a compact particle. Therefore, its potential energy periodically decreases and finally reaches the much lower level than the (001) nanoplate. However, the saddle surface of the (001) nanoplate demonstrated in Fig. 1b retains until it becomes irregular particle at about 200 K. The evolution of this structure with medium structural stability is accompanied by relatively mild energy change, which can be divided into four stages as demarcated by dot lines in the plots of potential energy.

To quantitatively investigate the morphology variation, we fitted the middle layer of (001) nanoplate by the quadric surface equation *z* = a*x*^2^ + b*y*^2^ + c*xy* + d. The fitting results are provided beneath the energy plots in Fig. 1, in which a, b, and c are the geometric parameters in the equation, and *R*^2^ refers to the coefficient of determination. *R*^2^ indicates the fitting degree, and its ideal value equals to 1. In accordance with the energy variations, the corresponding transitions of these fitting parameters are also observed at the critical points between different stages. The critical temperatures are identified to be 8, 129, and 205 K. During the first three stages, the value of *R*^2^ stays higher than 0.8, implying that the (001) nanoplate approximately keeps its saddle shape. Since the parameter c is obviously larger than both a and b, the two raised sections of the nanoplate are oriented along the [110] direction. Meanwhile, the value of c significantly increases after the first stage, which suggests the remarkably bending upwards surface. This tendency can be clearly perceived from the representative snapshots in Fig. 1d–f, which are respectively taken at 8, 60, and 160 K. Each bending deformation pulls the system out of its former metastable state and results in a slight drop in the potential energy. These minor adjustments of shape and energy end at 205 K, from where the fourth stage starts and the original saddle surface gradually collapses into an irregular particle with further minimized energy.

**Fig. 2:**
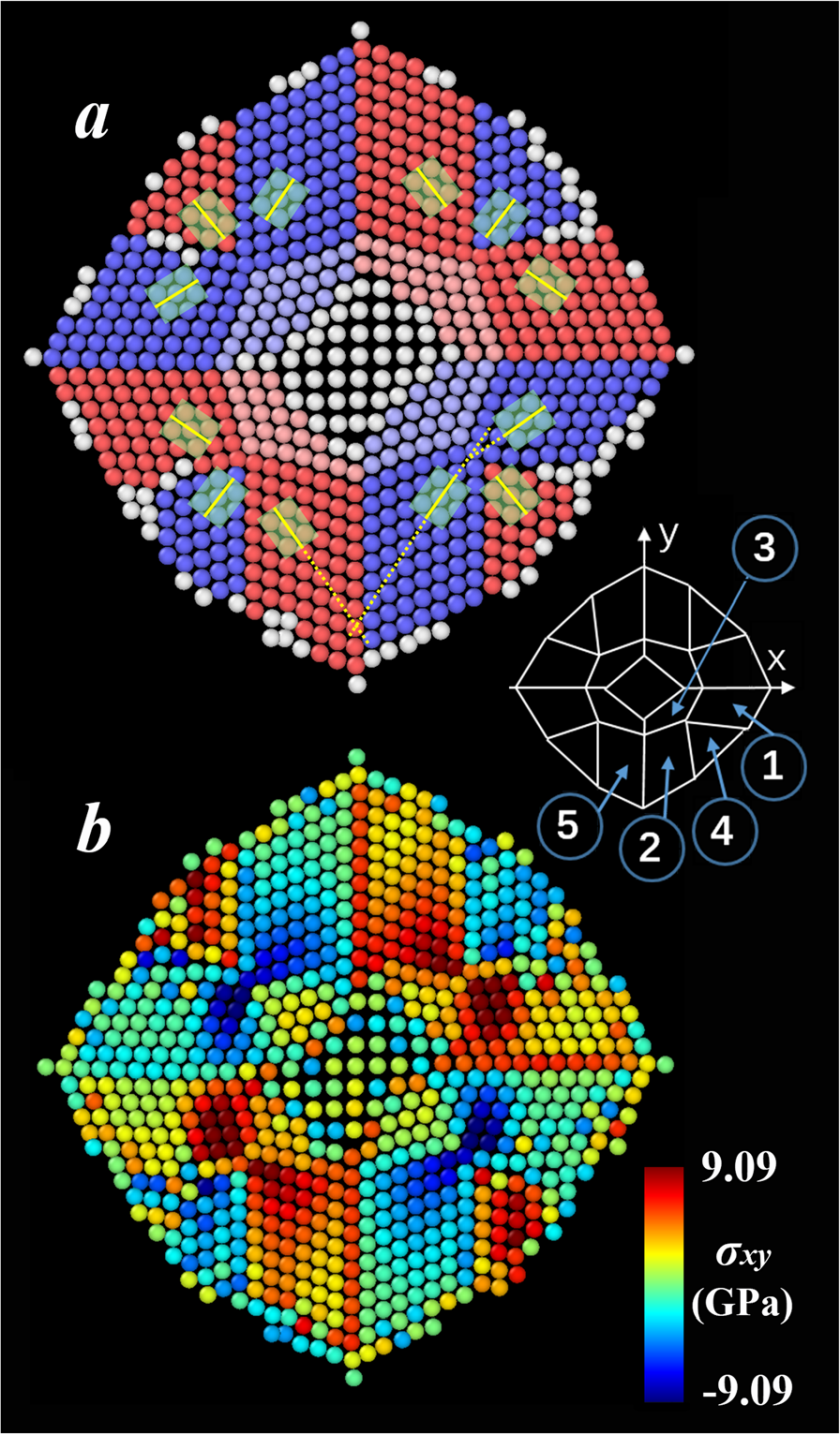
**a** Snapshot of the upper surface of (001) nanoplate after relaxation at 1 K and **b** the corresponding shear stress distribution

To examine the deformation mechanism of the (001) nanoplate in details, we investigated the atomic arrangements and the stress distribution. After relaxation at 1 K, the potential energy of the nanoplate is released to a great extent by structure bending along the [110] direction, as discussed above. During the formation of this metastable state, no inter-layer diffusion of atoms is observed. Figure 2a presents the vertical view of its upper surface. Note that the situation in the other two atomic layers essentially resembles the one described in the following. From the analysis of the lattice structure, most atoms (except the ones colored in white) are identified to form (110) facets, i.e., the initial (001) lattice transforms into the most close-packed structure in bcc crystal and the reconstruction occurs. In Fig. 2a, atoms in the adjacent facets are assigned to different colors. The unit cell of each facet is labeled by a green rectangle, in which the short yellow line indicates its respective [110] direction. As can be seen, these (110) facets, which are schematically illustrated in the lower right corner of Fig. 2a, are arranged in different orientations. The distribution is roughly in a symmetric fashion. Take one-fourth part of the total surface as an example, facets 1 and 2 basically align in parallel, and they are approximately perpendicular to facets 4 and 5. The atoms in facet 3 are slightly distorted to accommodate the lattices of both facets 1 and 2.

From the analysis of the calculated stress in the nanoplate, it is found that the shear stress shows an apparent correlation to the deformation, which makes it distinct from other components of the stress tensor. Therefore, we consider the shear stress as the primary driven force of the bending deformation and present its distribution in Fig. 2b. Clearly, the stress is no longer uniformly distributed owing to the short-range order induced by the emergence of those (110) facets. From the comparison of Fig. 2 a and b, it is found that the stress distribution is highly correlated to the orientation arrangement of the facets. The stresses in the red facets shown in Fig. 2a are generally positive, while they are negative in the blue ones. This observation suggests that the red and blue facets roughly tend to move towards the opposite directions. Significant stress gaps are observed to exist near the grain boundaries. These accumulated stresses are accompanied with the formation of (110) facets and would be released through further deformation of the nanoplate.

**Fig. 3:**
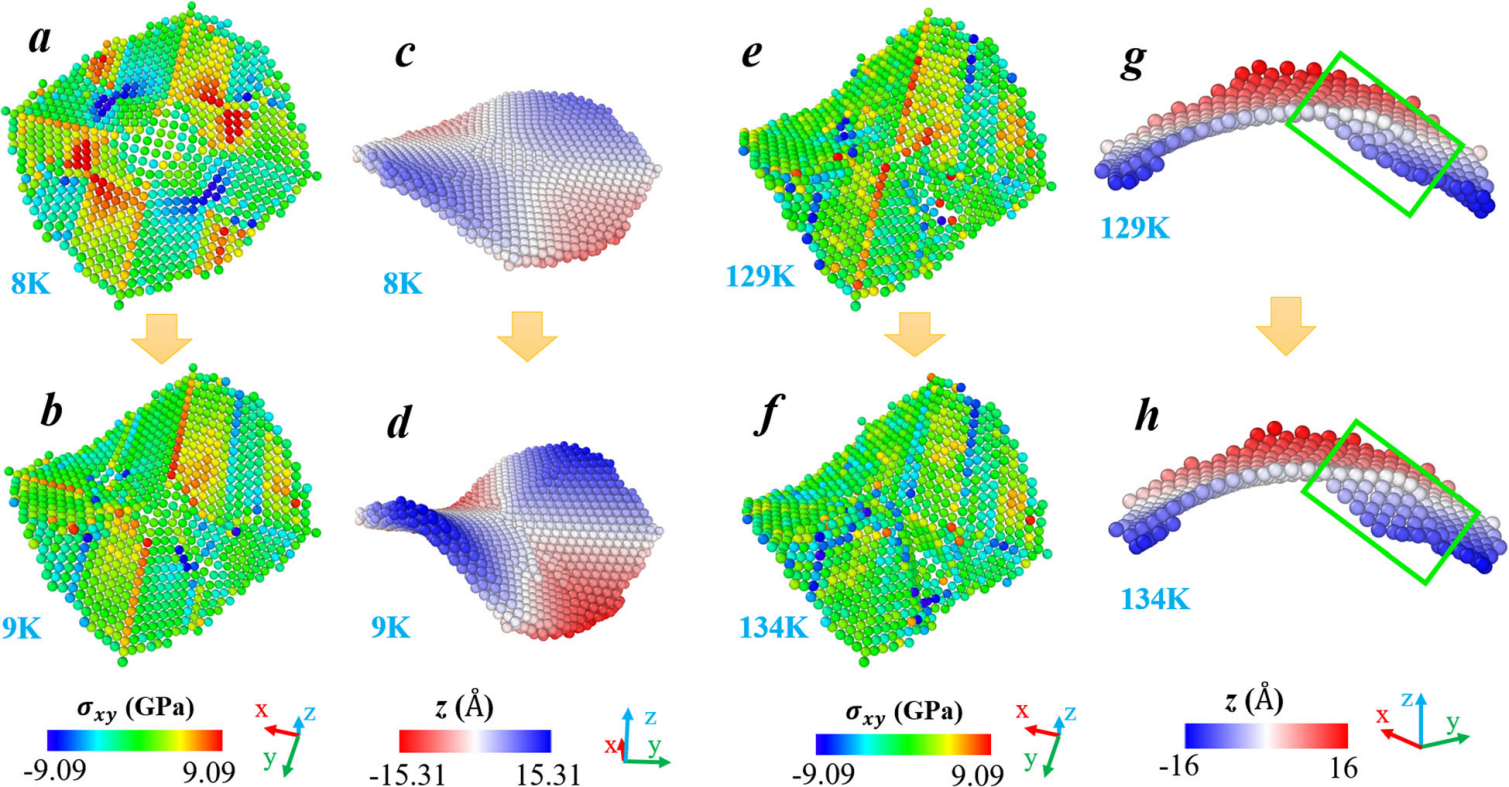
Two critical transformations during the temperature evolution of the (001) nanoplate. For each state, the atoms are respectively colored according to **a**, **b**, **e**, **f** their values of shear stress and **c**, **d**, **g**, **h** *Z* coordinates

Figure 3 shows two critical transformations during the subsequent deformation of the (001) nanoplate. In order to be consistent with Fig. 2, we only illustrated the atoms in the upper layer. Distributions of both stress and Z coordinates are presented. As seen from Fig. 3a, the stress distribution at 8 K resembles the situation at 1 K (cf. Fig. 2b), and the bending exists (cf. Fig. 3c and Fig. 1d). When the temperature increases to 9 K, the bending significantly develops, as seen in Fig. 3d. Meanwhile, one can find that the originally built-up stress gaps disappear (comparing Fig. 3 a and b). The release of shear stress is resulted from this further bending deformation. Accordingly, the potential energy decreases (cf. Fig. 1), and the nanoplate evolves into a more stable state. As for the second transition shown in Fig. 3, it starts at 129 K and finishes at 134 K, experiencing a broader temperature range. Note that after the transition occurring at 9 K, considerably large positive stress still exists in the middle area of the surface (cf. Fig. 3b). Actually, this state of stress maintains throughout the entire second stage of the shape change process (9–129 K) (cf. Fig. 3e). Similarly, it is also the driving force of the following transition. Afterward, as seen in Fig. 3f, those red atoms in Fig. 3e turn green (or blue), indicating that the existing positive stress is released. For the purpose of highlighting the shape transformation happening between 129 and 134 K, only half of the atoms in the upper surface are exhibited in Fig. 3 g and h, in which the green box singles out the changed area. The region in the green box buckles toward −*Z* direction, leading to the deviation from the former saddle shape. This deviation could also be confirmed by the obvious drop of *R*^2^ value in Fig. 1.

**Fig. 4:**
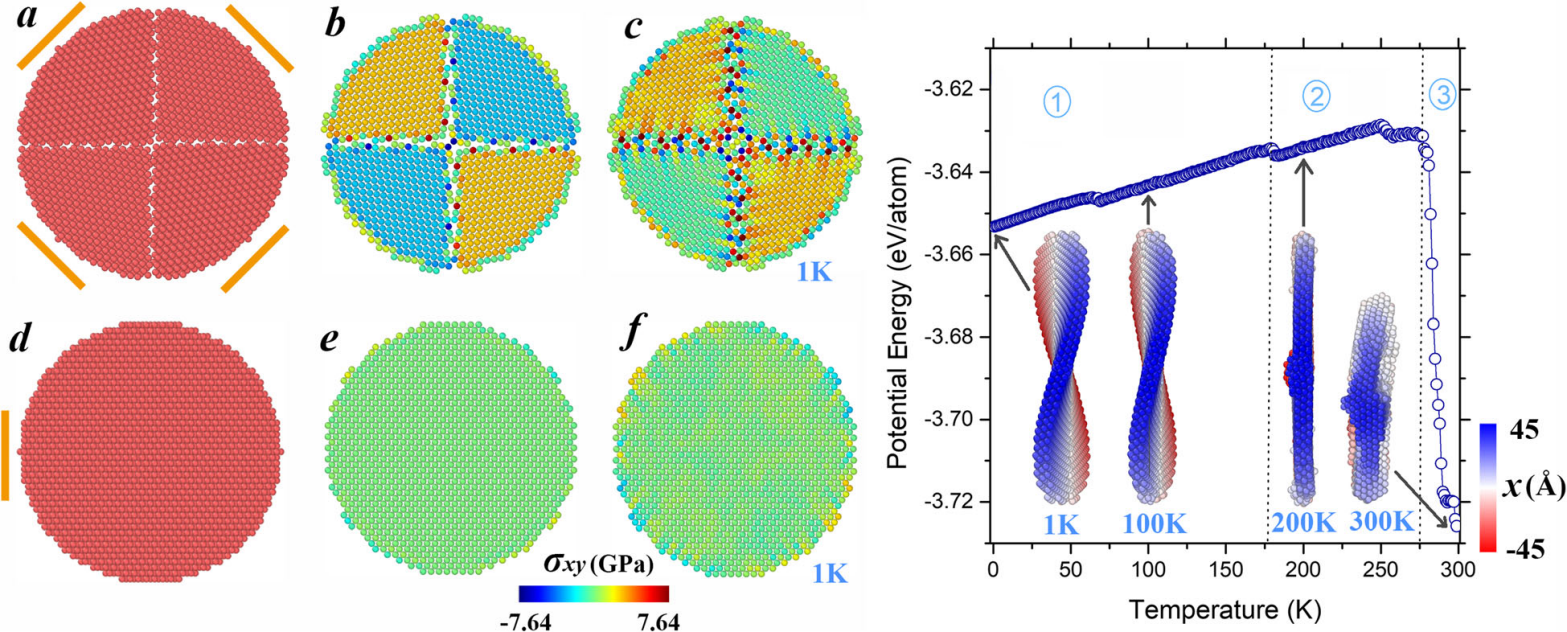
Left panel: Schematic illustration of **a** the modulated (110) nanoplate and **d** the regular one. **b**, **c**, **e**, **f** Respectively display their shear stress distributions in the initial configurations and at 1 K. Right panel: Temperature-dependent potential energies of the modulated (110) nanoplate. Snapshots in the insets are taken at representative temperatures

As is discussed above, the shape transformation of the (001) nanoplate is driven by the shear stress, whose distribution relies heavily on the lattice arrangement. In order to further exemplify the possibility of tailoring the morphology through crystalline orientation design, we modeled a nanoplate of three-layer (110) atoms, in which each layer is split into four different oriented facets, as demonstrated in Fig. 4a (referred as “modulated (110) nanoplate” hereafter). Orange lines in the schematic illustration indicate their respective [110] directions. To facilitate a comparison, we showed the regular (110) nanoplate in Fig. 4d. For the initial configuration, the calculated potential energy of the modulated (110) nanoplate equals − 3.617 eV/atom, higher than the corresponding value of the regular (110) nanoplate ( − 3.668 eV/atom) due to the existence of interfacial energy. In contrast to the uniform pattern of shear stress distribution of the regular (110) nanoplate (cf. Fig. 4e), remarkable stress gaps appear between adjacent facets in Fig. 4b. These gaps are especially significant among the atoms located near the grain boundaries. After relaxation at 1 K, the region with stress gradient extends to involve more atoms around the boundaries, as seen in Fig. 4c. Meanwhile, the averaged potential energy drops to − 3.653 eV/atom, and the configuration bending results in a saddle plate, similar to the (001) nanoplate. As the temperature is continuously elevated, during the shape evolution of the modulated (110) nanoplate, three stages can be identified with 179 and 277 K as the critical points. In the first stage, the saddle shape basically maintains despite minor fluctuations, as exemplified in the inset snapshot of 100 K. However, after the transition occurring at 179 K, the configuration converts back to be disk-like and keeps this shape without obvious change throughout the second stage (see, for example, the inset snapshot of 200 K). Around the critical point (179 K), note that the raised part in the center combined with the fragmented surface plane still corresponds to the configuration with lower energy. Finally, when the temperature reaches 277 K, the system begins to shrink to irregular particle (cf. the inset snapshot of 300 K), leading to the radical reduction in the potential energy, similar to the fourth stage of the (001) nanoplate described earlier. Note that the potential energy of the regular (110) nanoplate begins to drastically decrease at 552 K (the corresponding data points are not entirely presented in Fig. 1), the modulated (001) nanoplate shows significantly decreased shape stability. These results indicate that the design of crystalline orientations is an efficient approach to modulate the shape stability.

**Fig. 5:**
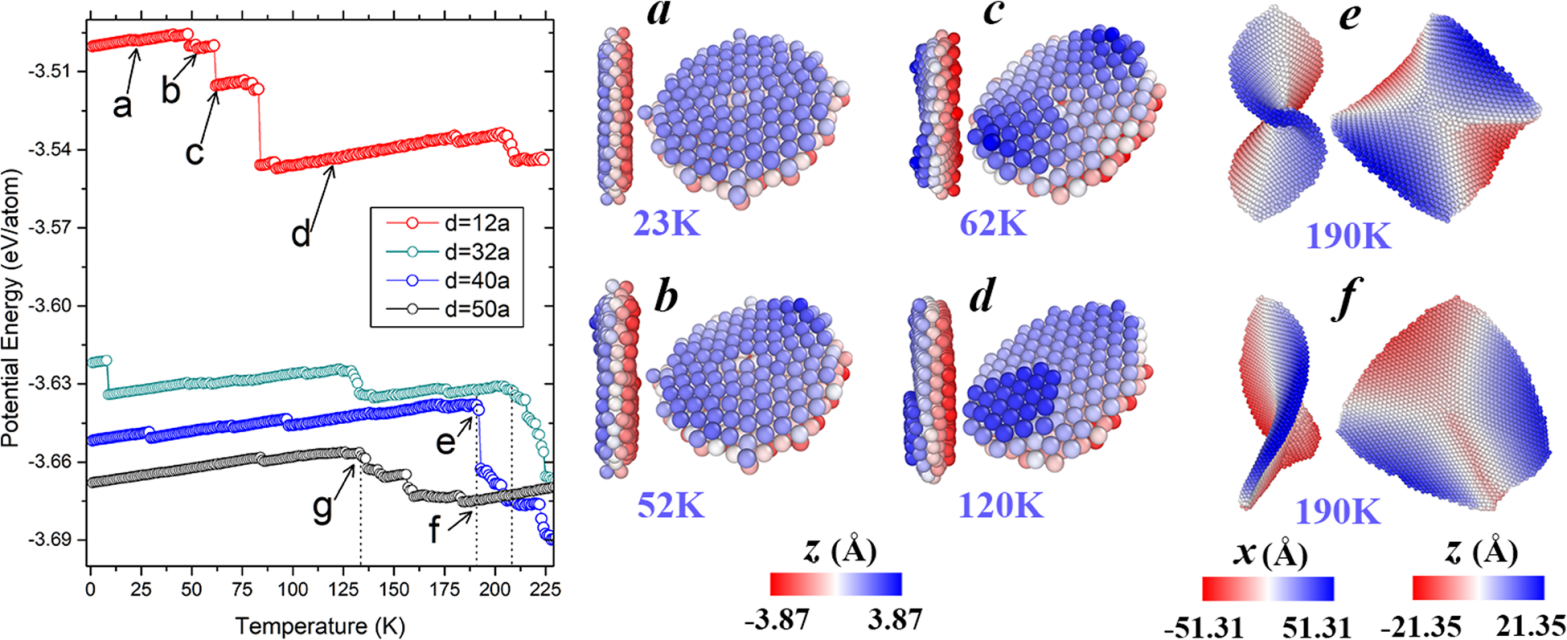
Left panel: Temperature evolutions of potential energies of bcc Fe (001) nanoplates with different diameters. Right panel: Snapshots of the nanoplate with *d* = 12*a* at **a** 23 K, **b** 52 K, **c** 62 K, and **d** 120 K; Snapshots of the nanoplate with **e** *d* = 40*a* at 190 K and (f) *d* = 50*a* at 190 K

To obtain a comprehensive understanding of the shape evolution patterns, we considered bcc Fe (001) nanoplates with different diameters (including *d* = 12*a*, 40*a*, and 50*a*). Their potential energies and typical configurations during the heating process are shown in Fig. 5. Note that the aforementioned nanoplate with *d* = 32*a* is also shown for comparison. With relatively high potential energy, the smallest nanoplate experiences more stages compared with the others. As seen in snapshot of Fig. 5a, the nanoplate with the diameter of 12*a* maintains its flat surfaces (a) until the bending starts to occur at 52 K (b) and finally the saddle shape forms at 62 K (c). However, this saddle structure does not last in a broad temperature range, and the following inter-layer diffusion happens at 84 K, leading to a sharp decrease in the potential energy. The thickened nanoplate, exemplified in Fig. 5(d), keeps its feature until further concentration appears at about 200 K. As for the nanoplate with *d* = 40*a*, the saddle shape is stably held at the temperatures ranging from 1 to 190 K before collapsing into a compact particle. In the case of the nanoplate with *d* = 50*a*, the saddle shape remains until 134 K (indicated by the arrow at “g” point) and then distorts to irregular structure, as illustrated in Fig. 5f. As can be seen, at 190 K, where the nanoplate with *d* = 40*a* just begins to collapse, the one with *d* = 50*a* has already completed its shape transformation from saddle to irregular. These observations suggest that, as the diameter increases from 12*a* to 40*a*, the temperature range where the saddle shape could be stable gradually becomes wider; however, when the diameter keeps growing (to 50*a*, for example), the stability of the saddle shape decreases to a certain extent. That is, although larger diameter leads to better structural stability (lower potential energy at ground state), it is not the only determining factor to affect the stability, influence from other aspects (such as kinetic and entropy effects) also plays an important role, especially when the nanoplate is large enough.

**Fig. 6:**
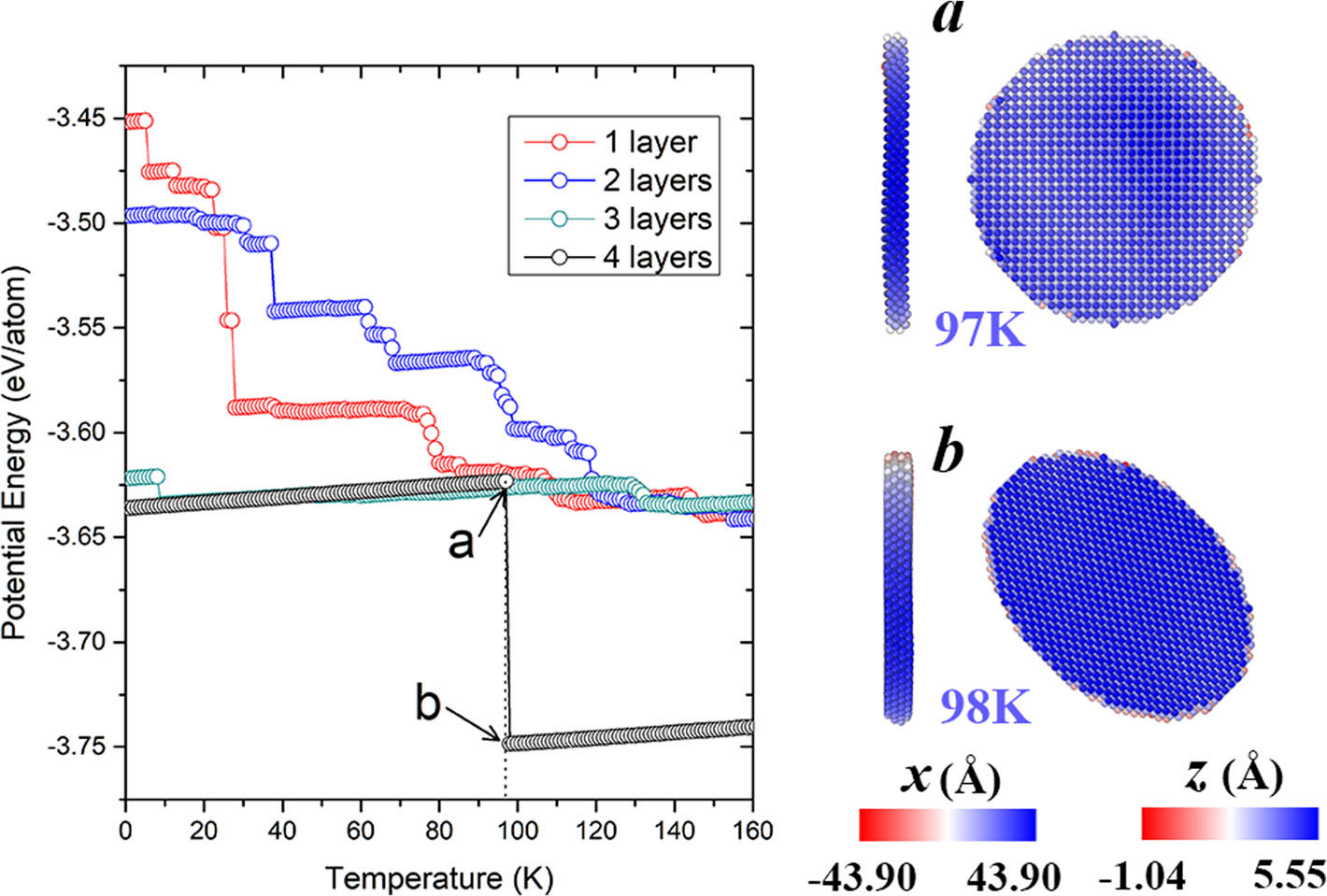
Left panel: Temperature evolutions of potential energies of Fe (001) nanoplates with different number of layers. Right panel: Snapshots of the nanoplate with 4 layers respectively at **a** 97 K and **b** 98 K

**Fig. 7:**
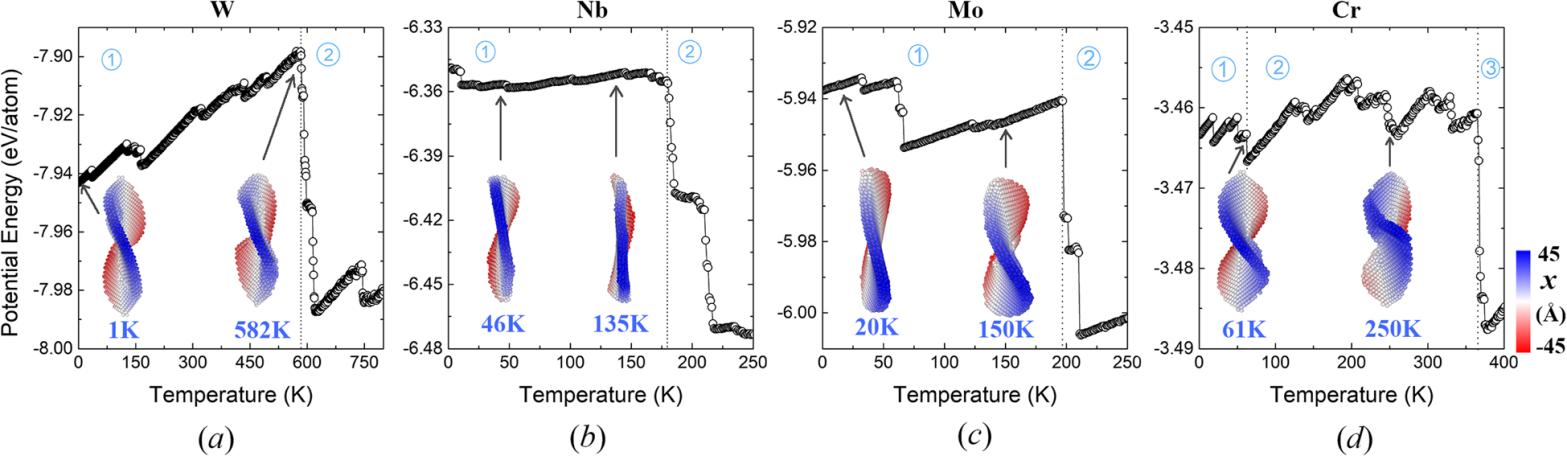
Temperature evolutions of potential energies of **a** W, **b** Nb, **c** Mo, and **d** Cr nanoplates respectively. Snapshots of the representative states are shown as insets

Besides the effect of diameter, nanoplates with different thicknesses are also considered. The potential energy of the nanoplate (*d* = 32*a*) with different layers are shown in Fig. 6. Note that the aforementioned 3-atomic-layer nanoplate is also shown for comparison. As seen from the evolution of the potential energies, it is found that the nanoplates with 1 or 2 atomic layers experience obviously more stages comparing to the one with 3 layers. Actually, they collapse to irregular shapes at much lower temperatures. In contrast, the original structure of the nanoplate with 4 layers is well maintained until 97 K (cf. Fig. 6a). However, at 98 K, its disc-like (001) planes transform into elliptical (110) planes with higher stability (cf. Fig. 1), which is accompanied with abrupt decrease in potential energy. This generated 4-layer (110) nanoplate retains its configuration until melting occurs. These results show that thicker nanoplates generally present better stability, and the saddle shape only exists at relatively small thicknesses. For further insights into the generality of the evolution patterns, we also modeled several other bcc metallic (including W, Nb, Mo, and Cr) nanoplates with diameters of 32*a*, which consist of three layers of (001) orientated atoms initially. Figure 7 illustrates the temperature-dependent potential energies of these nanoplates and the associated atomistic snapshots at representative temperatures. After relaxation at 1 K, all the originally uniform (001) planes reconstruct and form (110) facets with different orientations. At lower temperatures, the saddle shape, as a universal metastable state, appears in every nanoplate, similar to the case of Fe nanoplate. With the heating progressing, the transformation into an irregular particle occurs at different temperatures, where the potential energies sharply decrease. Comparatively, the saddle shape stage for W nanoplate lasts in the widest temperature range (until 582 K), which is attributed to its extraordinary structural stability (initial *E*_p_ = − 7.94 eV/atom). In contrast, the least stable Cr nanoplate maintains its saddle shape only until 62 K, after which the bumping and buckling successively appear (cf. the snapshots taken at 61 and 250 K in Fig. 7). As for the other two nanoplates, Nb tends to restore the original flat surface (cf. the snapshot of 135 K in Fig. 7), and Mo presents significant bending (cf. the snapshot of 150 K) before their final collapse. These two situations roughly resemble the modulated (110) and (001) Fe nanoplates. The above results show that the metastable states observed in the Fe nanoplates also exist in other bcc metallic nanoplates. Configurations with different structural stability follow different evolution patterns. In addition, it is noted that in most of our simulations, nanoplates transform into compact particles even below the room temperature, which is resulted from their small sizes. Still, the identified evolution mechanisms are of general significance. The results of the relative stability of these nanoplates between different plane orientations, sizes, and elements could be extrapolated to larger systems. The description of shape transformation mechanisms can serve as a reference for obtaining desired morphologies through crystalline orientation controlling or alloying [31, 32].

## Conclusions

In summary, the shape evolution of bcc Fe nanoplates with low-index surfaces was investigated by MD simulations. The discrepancy in structural stability leads to diverse patterns in morphology transformations. (110) nanoplate is the most stable and keeps its initial configuration until the highest temperature. By contrast, (111) and (001) nanoplates are not able to firmly exist, both of them tend to collapse into irregular particles even below the room temperature. However, prior to this final collapse, the surface of (001) nanoplate converts into (110) facets with different orientations and meanwhile forms a saddle shape, which maintains in a relatively broad temperature range. This transformation process is driven by the shear stress, whose distribution is closely associated with the facet arrangement. Further bending and buckling during the subsequent heating correspond to the stress release. Moreover, simulations were performed on the modulated (110) nanoplate, (001) nanoplates with different diameters and thicknesses, and other bcc metallic (001) nanoplates. The results show that the shape evolution can be tuned by facet orientations, plate sizes, and components. This study discloses the atomic-level mechanism of shape evolution of bcc metallic nanoplates and thus provides a theoretical basis on the morphology controlling in syntheses of metallic nanomaterials.

## Data Availability

All data generated or analyzed during this study are included in this published article.

## Abbreviations

a: Lattice constant
bcc: Body-centered-cubic
d: diameter
EAM: Embedded atom method
*E*_p_: Potential energy
MD: Molecular dynamics
*R*^2^: Coefficient of determination
TEM: Transmission electron microscope
